# State of the Science of Endocrine Disruptors

**DOI:** 10.1289/ehp.1306695

**Published:** 2013-04-01

**Authors:** Linda S. Birnbaum

**Affiliations:** Director, NIEHS and NTP, National Institutes of Health, Department of Health and Human Services, Research Triangle Park, North Carolina, E-mail: birnbaumls@niehs.nih.gov

On 19 February 2013, the United Nations Environment Programme (UNEP) and the World Health Organization (WHO) released *The State of the Science of Endocrine Disrupting Chemicals - 2012* (WHO/UNEP 2013), an extensive update of an earlier document, *Global Assessment of the State-of-the-Science of Endocrine Disruptors* (International Programme on Chemical Safety 2002). Over the past decade there have been significant advances in our understanding of endocrine disrupting chemicals (EDCs): their numbers, mechanisms of action, biological effects, and impacts on human and wildlife health.

First, as described in the new report (WHO/UNEP 2013), the convergence of wildlife, laboratory animal, and epidemiology data suggests a greater role for EDCs in disease, even more than was predicted just 10 years ago. Taking the animal and human evidence together, the report demonstrates a strong likelihood that exposure to EDCs during fetal life and/or puberty plays a role in the proliferation of male and female reproductive problems, endocrine-related cancers, infections, asthma, obesity, diabetes, and behavioral and learning disorders, including attention deficit/hyperactivity disorder (ADHD).

The incidences of these conditions have increased significantly not only in the United States but across the globe. Because genes do not change fast enough to explain this increase, environ-mental causes must be involved. The environmental contribution to disease is estimated to be 24–33% of the global disease burden (Smith et al. 1999). Although it is challenging to address the role of the environ-ment in disease, there is also a tremendous opportunity to improve human health by identifying environ-mental elements that affect public health. The recog-nition of these challenges and opportunities, along with the fact that many diseases are associated with the endocrine system (e.g., infertility, diabetes, breast and prostate cancer) has led to the focus on EDCs.

Second, it is now accepted that development (*in utero* and the first years of life) is a very sensitive time for EDC-induced health effects (Kortenkamp et al. 2011). Over the last 10 years, the focus of EDC research has shifted from investigating adult exposure and disease outcomes to examining develop-mental exposure and later-life disease outcomes. This latter approach is now considered the most appropriate approach for most endocrine-related diseases/disorders (U.S. Environmental Protection Agency 2012).

Third, identifying those chemicals with endocrine activity from all the chemicals used and released worldwide is a major challenge, and we are likely assessing only the “tip of the iceberg.” Hundreds of chemicals, as well as persistent organic pollutants, have been identified as EDCs. EDCs are not uniform: They have very different properties, sources, and fates in the environment. Although it may be possible to trace high production volume chemicals, numerous additives and process chemicals are not traceable. Adding to this complexity are the unintended and possibly unknown by-products of chemical manu-facturing, including chemicals resulting from combustion, toxicants resulting from the transformation of chemicals after their release into the environment, and internally created metabolites, all of which may increase the number of potential exposures to EDCs in our environment.

EDCs likely affect all hormonal systems that control the develop-ment and function of reproductive organs, regulation of metabo-lism, and satiety. Recent research has shown that EDCs also affect physiological systems that control fat develop-ment, weight gain, and glucose levels (Thayer et al. 2012).

Finally, as noted in the new WHO/UNEP report (WHO/UNEP 2013), EDCs are a global problem and will require global solutions. EDCs have contaminated the world via the natural flow of air and water. Several hundred EDCs have been measured in humans and wildlife, even in remote places such as the Arctic. Thus, it is now impossible to examine an -unexposed population anywhere on Earth. To improve health, environmental health scientists and toxicologists need to work more closely with colleagues in endocrinology, genetics, developmental biology, epigenetics, and clinical medicine to bring EDC research into the mainstream of science.

These and other important topics related to EDCs and their effects are detailed in *The State of the Science of Endocrine Disrupting Chemicals - 2012* (WHO/UNEP 2013). I commend WHO/UNEP and the scientists who worked for more than 2 years to produce this report. It is an important source of data on EDCs and should be mandatory reading for everyone who is interested in protecting and improving human health.
